# Parasitic Infections in Internationally Adopted Children: A Twelve-Year Retrospective Study

**DOI:** 10.3390/pathogens11030354

**Published:** 2022-03-15

**Authors:** Elena Chiappini, Teresa Paba, Matilde Bestetti, Luisa Galli

**Affiliations:** 1 Pediatric Infectious Disease Unit, Anna Meyer Children’s University Hospital, 50100 Florence, Italy; teresa.paba@stud.unifi.it (T.P.); matilde.bestetti@stud.unifi.it (M.B.); luisa.galli@unipi.it (L.G.); 2 Department of Health Sciences, University of Florence, 50100 Florence, Italy

**Keywords:** intestinal parasitosis, paediatrics, *Toxocara canis*, *Giardia lamblia*, *Entamoeba coli*, *Strongyloides* spp., eosinophilia, screening

## Abstract

Parasitic infections (PIs) are among the most frequent infectious diseases globally. Previous studies reported discrepant results regarding the prevalence of PIs in internationally adopted children (IAC). Data from IAC referred to our paediatric university hospital in 2009–2021 were collected to evaluate the frequency of PIs by the use of stool microscopic examination, antigen assays for *Giardia lamblia* and *Cryptosporidium parvum*, and serological tests for *Toxocara canis*, *Strongyloides stercoralis*, *Schistosoma mansoni*, *Echinococcus* spp., *Taenia solium*, and *Trypanosoma cruzi*. Uni- and multivariate logistic regression analyses were performed to evaluate risk factors for PIs and eosinophilia. The proportion of IAC with at least one positive test was 26.83% (640/2385); 2.13% (*n* = 51) had positive tests for 2 or 3 parasites. A positive assay for helminthic infection was retrieved in 11.07% of children (*n* = 264), and 17.86% (*n* = 426) presented with eosinophilia. The most common positive tests were anti-*Toxocara canis* antibodies (*n* = 312; 13.8%), followed by positive stool antigen for *Giardia lamblia* (*n* = 290; 12.16%)*,* and positive microscopic stool examination for *Blastocystis hominis* (*n* = 76; 3.19%). A statistically significant association was found between PIs and region of origin (children from Latin America and Africa were more likely to present PIs than children from Eastern Europe), age 5–14 years, and eosinophilia. No significant association was observed between PIs and gender, vitamin D deficiency, or anemia. In conclusion, PIs are relevant in IAC and an accurate protocol is needed to evaluate IAC once they arrive in their adoptive country.

## 1. Introduction

Parasitic infections (PIs) are among the most frequent infectious diseases globally [1]. These ‘neglected tropical diseases’ generally harbour deplorable hygienic conditions and unfavourable social and climatic conditions [1,2]. Unfortunately, internationally adopted children (IAC) are a population that is often hit by this spectrum of conditions and, therefore, must be carefully assessed.

In Italy, around 2000 children are adopted by Italian families every year, with Tuscany ranking second after Lombardy [3]. However, the characteristics of the population of IAC are difficult to define: extraordinarily diverse and heterogeneous, often coming from deprived contexts and countries where several infections are endemic [4].

It is crucial to assess the health state of IAC once they arrive in their adoptive country. Preadoptive records are often scarcely reliable, and children often declared healthy in their region of origin might be affected by several diseases [5]. The immunisation status is often uncertain. Moreover, they have often lived in orphanages or other centres that can increase the risk of infectious diseases [5,6]. Therefore, it is important to diagnose possible morbidities, infectious and not, that can seriously impact the child’s health [7,8].

In previous studies, the reported PI prevalence greatly varied ranging between 9% and 72% [1,9,10,11]. In general, a trend toward a higher prevalence may be observed in studies dating back 10 years or less [1,9], with respect to the older ones [10,11]; improvements in diagnostic techniques over time can partially explain these differences. 

The starting point of the assessment should be the screening of the child’s health [1,5]. In our centre, according to the guideline recommendations [5], IAC routinely undergo serology testing for the most common parasites, microscopic stool examinations, and stool assays for *Giardia lamblia* and *Cryptosporidium parvum* antigens [12]. Additional tests may be performed in patients with an increased risk for peculiar conditions, such as serologic testing for Chagas, schistosomiasis, or filariasis. We previously reported that PIs are the most frequent infectious diseases diagnosed in IAC, followed by skin infections and latent or active tuberculosis [12,13].

The management of IAC lacks evidence-based guidelines and fails to cover the entirety of the conditions that might affect the population [14]. Eosinophilia can be linked to parasitosis [15,16]. Other clinical conditions possibly associated with PIs include malnutrition, anemia, and vitamin D deficiency [17,18,19].

It is essential to detect possible parasitosis as soon as possible, in order to avoid severe long-term consequences, such as growth and developmental delays, and behavioural problems [20,21,22,23]. Moreover, in the current literature, there are insufficient or contrasting data regarding the prevalence of the PIs in IAC according to their birth countries or age [1]. The aim of this study is to evaluate the prevalence of and possible risk factors for PIs in a large cohort of IAC.

## 2. Results

In the study period, 2385 IAC were enrolled. Overall, 1420 (59.5%) children were male, median age was 5.78 years (95%CI: 3.28–8.17). The majority of the children originated from Eastern Europe (40.04%), followed by Latin America (22.18%), Asia (20.63%), and Africa (17.15%). In particular, the most common countries of origin were Russia (*n* = 534; 22.39%), Colombia (*n* = 187; 7.84%), India (*n* = 182; 7.63%), Ethiopia (*n* = 163; 6.83%), Hungary (*n* = 133; 5.58%), Vietnam (*n* = 112; 4.70%), Brazil (*n* = 108; 4.53%), Congo (*n* = 96; 4.03%), and Chile (*n* = 90; 3.77%).

The majority of the children were between 1 and 9 years of age, representing 89% of the study population; among them, 984 IAC (41.26%) had Vitamin D deficiency; eosinophilia was found in 17.86% (*n* = 426) of the children (Table 1).

Overall, 640 (26.83%) IAC tested positive for a least one parasite, including 48 (2.01%) children with positive tests for 2 parasites, and 3 (0.12%) children positive for 3 parasites. A positive assay for helminthic infection was retrieved in 11.07% of children (*n* = 264).

The most common positive tests were anti-*Toxocara canis* antibodies (*n* = 312; 16.44%*)*, followed by positive stool antigen for *Giardia lamblia* (*n* = 290; 12.16%) and positive microscopic stool examination for *Blastocystis hominis* (*n* = 76; 3.19%) (Table 2).

Positive tests for PIs were more frequently observed in children aged 5 to 14 years (*p* < 0.0001) (Table 3). A positive serologic test for *Toxocara canis* was the most common positive assay in all age groups, except for the children between 1 to 4 years of age for whom a *Giardia lamblia* positive test was the most frequent positive assay (14.12%) (Table 3).

Latin America was the region of origin with the highest percentage of positive tests for parasites (185/529; 34.97%; *p* < 0.0001 vs. Eastern Europe), followed by Africa (126/409; 30.81%; *p* < 0.0001 vs. Eastern Europe), Asia (125/492; 25.41% *p* = 0.112 vs. Eastern Europe) and Eastern Europe (204/955; 21.36%) (Table 4; Table S1).

At univariate and multivariate analyses, factors significantly associated with a positive assay for parasites were eosinophilia (*p* < 0.0001), age groups of 5–9 years (*p* < 0.0001) and 10–14 years (*p* = 0.028), and origin from Africa or Latin America (*p* < 0.0001 vs. Eastern Europe) (Table S1). We observed that a higher risk for a positive test for parasites was present in IAC with severe eosinophilia, compared with those with mild eosinophilia (Table S1). Gender, vitamin D levels, and anemia were not significantly associated with a positive assay for parasites.

Analysis exploring the risk factors for eosinophilia confirmed a significant association with a positive test for parasites (OR: 2.04; 95%CI: 1.63–2.53; *p* < 0.0001), and an increased risk in children originating from Latin America (*p* = 0.038 vs. Eastern Europe) or Asia (*p* < 0.001 vs. Eastern Europe) (Table 3). In particular, IAC with a positive test for helminths (OR: 2.06; 95%CI: 1.39–3.05; *p* < 0.0001) or those with positive tests for both helminths and protozoa (OR: 2.74; 95%CI:1.45–5.02; *p* = 0.001) were more likely to present eosinophilia than children with no positive test or those positive only for protozoa (Table S2).

## 3. Discussion

In the present study, 2385 IAC referred to one university centre were prospectively enrolled from 2009 to 2021. A positive test for parasites was observed in 26% of the study children, and 17.86% of the study children had eosinophilia, whose correlation with parasitic infections has been proven by multiple studies [1,10,16].

The most common positive tests were specific IgG anti-*Toxocara canis* (*n* = 312; 16.44%*)*, followed by positive stool antigen for *Giardia lamblia* (*n* = 290; 12.16%)*,* and positive microscopic stool examination for *Blastocystis hominis* (*n* = 76; 3.19%).

A statistically significant association was found between PIs and region of origin (children from Latin America or Africa were more likely to present a positive test than children from Eastern Europe or Asia), age in the range of 5–14 years, and eosinophilia. The risk increased, as the eosinophilia was more severe. No significant association was observed between the prevalence of PIs and gender, vitamin D deficiency, and anemia.

The previously reported prevalence of PIs in IAC ranges greatly, depending on the age of the child and country of origin. In one of the largest studies, at least one intestinal parasite was detected in 27% of 1042 IAC [11], while in a large French study, the prevalence reached 35% [24].

In other observational studies, the rates of recovery of parasites ranged from 9% to over 70% [24,25,26,27,28,29].

The prevalence was similar in children with and without gastrointestinal symptoms and with and without malnutrition. In most studies, giardiasis was the most commonly identified parasitic infection, with as many as 19 percent of children infected [24,25,26,27,28,29]. Helminthic infections are less common (<3 percent), with *Hymenolepis species*, *Ascaris lumbricoides*, and *Trichuris trichiura* most often reported.

Different population characteristics and used laboratory techniques partially explain the different prevalence of parasites reported by several studies. In agreement with other studies, a statistically significant relation between anemia and PIs was not observed in our study [1,10,11]. This lack of association could be due to the type of PIs observed among our study children, since helminthic infections, which are more commonly associated with intestinal bleeding and anemia [1], were a minority of PIs on our dataset.

Other authors reported that the PI prevalence was similar in children with and without gastrointestinal symptoms [10], with and without malnutrition [10], or anemia [1], suggesting that the decision to screen for intestinal parasites should not be based upon symptoms or nutritional status of IAC. 

Our results confirm that not all PIs are associated with eosinophilia. Although an increase in peripheral blood count is more frequently associated with helminthic infections [30,31,32,33], this usually occurs when the parasite invades the host’s tissues. Widespread parasitosis such as *Giardia lamblia* infection, which our study reported affecting 12.6% of the IAC, are intraluminal and, therefore, are not associated with eosinophilia [1]. On the other hand, in IAC the risk of PIs is substantial, as only 14.6% of children with eosinophilia had all the test negative. Accordingly, given this high risk, some authors have suggested starting a presumptive treatment with albendazole in IAC with eosinophilia despite negative parasitic test results [10].

One of the limitations of our study is its retrospective study design. Moreover, a positive serologic result may not always correspond to a PI actually present at the moment of the investigation and results should be interpreted cautiously. Finally, the analyses were performed considering regions of origin and not every country, since the numbers were too dispersed in small subgroups to carry out the analyses by country of origin. Morevoer, the considered regions were very extensive and had a great diversity of eco-epidemiological scenarios.

In conclusion, our study found the prevalence of PIs to be relevant in IAC, especially in those aged 5–14 years and originating from Africa or Latin America. A protocol including both a parasitological exam and serologic testing is needed to evaluate IAC once they arrive in their adoptive country.

## 4. Materials and Methods

### 4.1. Study Design

This retrospective study was performed at the Anna Meyer Children’s University Hospital in Florence, enrolling all the IAC <18 years old (originating from any foreign region of origin), who underwent the internal operative protocol for the first screening in a 12-year period (January 2009 to December 2021), as previously described [5]. Children adopted from Italy were excluded from the study. The screening was offered to Tuscan adoptive families who were referred to the Centre for the Internationally Adopted Child and consented to the evaluation.

### 4.2. Data Collection

Data were prospectively collected and entered into an electronic database following international standards for data protection, including a precise review of all the following documentation presented during the first visit: family and personal medical history, immunisation status, the results of laboratory tests, and a clinical evaluation. The following data were entered into the database and analysed:

1. Gender, age, and region of origin;
2. Serum vitamin D concentration;
3. Haemoglobin serum level (g/L);
4. Eosinophil blood cell count (cell/µL);
5. Results of parasitic stool examination;
6. Results of antigen assays for *Giardia lamblia* and *Cryptosporidium parvum* on stool samples;
7. Results from serological investigations for *Toxocara canis*, *Strongyloides stercoralis*, *Schistosoma mansoni*, *Echinococcus granulosus*, *Taenia solium*, *Trypanosoma cruzi*, performed in children at high risk of infection, according to their area of origin.

### 4.3. Laboratory Investigations

During the first evaluation, all the children underwent a venipuncture and laboratory assessment. All the laboratory examinations were executed in the same laboratory, following standardised techniques.

### 4.4. Samples Collection

Three stool samples were collected on alternate days in the lapse of 10 days.

The enrolled children and their parents received appropriate information regarding the correct procedure for faecal samples collection. Subsequently, they were asked to fill stool transport vials ParaPak EcoFix (Meridian Bioscience, Cincinnati, OH, USA) containing fixative medium, with faecal samples. Each sample was placed on a different vial and preserved at 4 °C until their transport to the laboratory. Microscopic analysis of the faecal material was performed daily.

### 4.5. Coproparasitological Test

Microscopic analysis for enteric protozoan cysts and/or trophozoites was performed according to the standardised procedures with the following methods: Concentration of samples—each faecal sample was concentrated by means of the commercial filter SpinCon (Meridian Biosciences, Cincinnati, OH, USA), which employs passive filtration and centrifugation through a series of two screens with successively smaller mesh. For the process of filtration 3 mL of surfactant treated, preserved stool specimens were manually transferred from their transport vials to the device for filtration. The surfactant consented to break down faecal aggregates, thus helping to release the parasites. This was followed by the physical blending of faecal material and the addition of 2 mL of physiological saline (for a total of approximately 5 mL diluted, filtered stool), to facilitate the concentration process. The faecal samples were then centrifuged at 500× *g* for 10 min, and the supernatant was eventually discarded into a suitable biohazard receptacle, thus producing a small pellet of a concentrated sample, which was examined microscopically for the presence of parasites. Direct microscopic analysis with extemporary staining was carried out, during which a small quantity of faecal material (about 2 mg) was diluted on a glass slide with Lugol’s iodine solution 1% (containing iodine crystals 2 g, potassium iodide 2 g, and distilled water 100 mL) stabilised with polyvinylpyrrolidone (PvPP). The specimen was then analysed using a low-power lens (10×) and low-intensity light by means of Kohler lighting. The ambiguous samples were further observed at 40×. *Antigen detection* was performed by immunochromatographic test (Stick Crypto/Giardia; Operon^®^, Zaragoza, Spain) according to the manufacturer’s instructions

### 4.6. Serological Investigations

Serum samples were tested for specific antibodies using commercial immunoenzymatic assays according to the manufacturer’s instructions. Serologic tests were performed to detect specific IgG antibodies (*Toxocara canis*, *Strongyloides stercoralis*, *Schistosoma mansoni*, *Echinococcus granulosus*, *Taenia solium*, and *Trypanosoma cruzi*). For the diagnosis of *Toxocara canis*, an additional Western blot serology was performed when the result of the serologic testing was uncertain. A single ELISA test was used for several infections (‘Filariasis ELISA kit’, Bordier Affinity Products SA, Crissier, Switzerland, for filariasis; ‘Schistosoma mansoni ELISA kit’, Bordier Affinity Products SA, for schistosomiasis; ‘Strongyloidiasis ELISA kit’ based on Strongyloides Ratti antigens, Bordier Affinity Products SA, for Stronglyloides; and ‘DRG Toxocara canis ELISA’, DRG Instruments GmbH, Marburg, Germany, for toxocariasis). The qualitative presence of antibodies for Trypanosoma cruzi (the aetiological agent of Chagas disease) was tested employing two ELISAs, one based on recombinant antigens (‘BioELISA Chagas’, Biokit, Lliça d’Almunt, Spain), the other based on crude antigens (‘BioELISA Chagas III’, BiosChile, Santiago, Chile) [33].

### 4.7. Definitions

Eosinophilia was defined as the elevation of eosinophil blood cell count (>500 cell/μL). Severe eosinophilia was defined as the elevation of eosinophil blood cell count >1000 cell/μL) [1].

Pathogenic/nonpathogenic parasites. Parasites were furtherly classified as pathogenic and nonpathogenic, according to the literature data [1,11]. Only pathogenic or potentially pathogenic parasites were evaluated.

### 4.8. Statistical Analysis

Data were reported as the median and interquartile range (IQR) or absolute numbers and percentages. All continuous variables were not normally distributed; thus, the nonparametric Mann–Whitney test was used to compare groups. Fisher’s exact test or chi-square test was used to compare categorical variables, as appropriate. Uni- and multivariate logistic regression analyses were performed to investigate the association between clinical and laboratory variables among groups (children with eosinophilia vs. children without eosinophilia; children with PIs (pathogenic, based on the results of the stool examination and serologic testing) vs. children with no infection; children with PIs (only pathogenic) vs. other enrolled children). Crude and adjusted odds ratios (ORs), with 95% confidence intervals (ICs), were estimated by performing, respectively, simple and multivariate logistic regression analyses. All variables that resulted from significant univariate analyses or were judged clinically relevant were included in the multivariate model.

All statistical analyses were carried out using the SPSS (Statistical Package of Social Sciences, Chicago, IL, USA) for Windows software program version 27.0. A *p* value < 0.05 was considered significant.

## Figures and Tables

**Table 1:** Personal characteristics, geographical origin, and laboratory test results of the study population (*n* = 2385).

Total Population	*n*	%
**Gender**		
Male	1420	59.54
Female	965	40.46
**Region of origin**		
Africa	409	17.15
Latin America	529	22.18
Asia	492	20.63
Eastern Europe	955	40.04
**Age Group**		
0–4 Years	1006	42.18
5–9 Years	1123	47.09
10–14 Years	227	9.52
15–18 Years	29	1.22
**Vitamin D**		
≤20 mg	984	41.26
>20 mg	993	41.64
Missing	408	17.1
**Anemia**		
No	1577	66.12
Yes	185	7.76
Missing	623	26.12
**Eosinophil cell count**		
<500 cell/µL	1915	80.29
500–1000 cell/µL	339	14.21
≥1000 cell/µL	87	3.65
Missing	44	1.84

**Table 2:** Results of investigations for parasites (by stool tests or serology) in the study population (*n* = 2385).

Protozoa	Children (*n*)	%	Helminths	Children (*n*)	%
*Giardia lamblia*	290	12.16	*Ascaris lumbricoides*	2	0.08
*Entamoeba histolytica*	7	0.29	*Hymenolepis nana*	21	0.88
*Entamoeba dispar*	2	0.08	*Ancylostoma duodenalis*	1	0.04
*Cryptosporidium parvum*	1	0.04	*Enterobius vermicularis*	7	0.29
*Blastocystis hominis*	76	3.19	*Strongyloides stercoralis*	12	0.5
*Dientamoeba fragilis*	34	1.43	*Toxocara canis*	312	16.44

**Table 3:** Results of investigations for parasites (by stool tests or serology) in the study population (*n* = 2385), by age class.

	1–4 Years		5–9 Years		10–14 Years		15–18 Years		Total	
	**1006**		**1123**		**227**		**29**		**2385**	
**Protozoa**	***n***	%	***n***	%	***n***	%	***n***	%	***n***	%
*Giardia lamblia*	142	14.12	133	11.84	14	6.17	1	3.45	290	12.16
*Entamoeba histolytica*	1	0.10	5	0.45	0	0.00	1	3.45	7	0.29
*Entamoeba dispar*	1	0.10	1	0.09	0	0.00	0	0.00	2	0.08
*Cryptosporidium parvum*	1	0.10	0	0.00	0	0.00	0	0.00	1	0.04
*Blastocystis hominis*	10	0.99	50	4.45	16	7.05	0	0.00	76	3.19
*Dientamoeba fragilis*	8	0.80	22	1.96	4	1.76	0	0.00	34	1.43
**Helminths**										
*Ascaris lumbricoides*	2	0.20	0	0.00	0	0.00	0	0.00	2	0.08
*Hymenolepis nana*	10	0.99	11	0.98	0	0.00	0	0.00	21	0.88
*Strongyloides stercoralis*	2	0.20	10	0.89	0	0.00	0	0.00	12	0.50
*Enterobius vermicularis*	2	0.20	5	0.45	0	0.00	0	0.00	7	0.29
*Toxocara* canis	61	6.06	202	17.99	46	20.26	3	10.34	312	13.08
*Ancylostoma duodenalis*	0	0.00	1	0.09	0	0.00	0	0.00	1	0.04

**Table 4:** Results of investigations for parasites (by stool tests or serology) in the study population (*n* = 2385), by region of origin.

Parasites According to Region of Origin										
	**Africa**		**Latin America**		**Asia**		**Eastern Europe**		**Total**	
	***n* = 409**		***n* = 529**		***n* = 492**		***n* = 955**		***n* = 2385**	
**Protozoa**	*n*	%	N	%	N	%	N	%	N	%
*Giardia lamblia*	103	25.18	84	15.88	48	9.76	55	5.76	290	12.16
*Entamoeba histolytica*	1	0.24	1	0.19	2	0.41	3	0.31	7	0.29
*Entamoeba dispar*	0	0.00	0	0.00	1	0.20	1	0.10	2	0.08
*Cryptosporidium parvum*	0	0.00	0	0.00	0	0.00	1	0.10	1	0.04
*Blastocystis hominis*	3	0.73	13	2.46	48	9.76	12	1.26	76	3.19
*Dientamoeba fragilis*	0	0.00	7	1.32	7	1.42	20	2.09	34	1.43
**Helminths**										
*Ascaris lumbricoides*	0	0.00	1	0.19	1	0.20	0	0.00	2	0.08
*Hymenolepis nana*	4	0.98	11	2.08	6	1.22	9	0.94	30	1.26
*Strongyloides stercoralis*	5	1.22	1	0.19	6	1.22	0	0.00	12	0.50
*Enterobius vermicularis*	0	0.00	3	0.57	1	0.20	3	0.31	7	0.29
*Toxocara canis*	22	5.38	108	20.42	39	7.93	143	14.97	312	13.08
*Ancylostoma duodenalis*	0	0	0	0.00	1	0.20	0	0.00	1	0.04

## Data Availability

Not applicable.

## Supplementary Materials

The following supporting information can be downloaded at: https://www.mdpi.com/article/10.3390/pathogens11030354/s1, Table S1: Univariate and Multivariate Analyses for risk factors for parasitic infections in the study population; Table S2: Univariate and Multivariate analyses for risk factors for a positive test result for at least one pathogen parasite.
