# Supplementary material for: Parasitic Infections in Internationally Adopted Children: A Twelve-Year Retrospective Study
Source: Pathogens. 2022 Mar 15;11(3):354. doi: 10.3390/pathogens11030354 (PMC8949827; doi:10.3390/pathogens11030354)
Supplement: Supplementary file 1 [file pathogens-11-00354-s001.zip › pathogens-1533286-supplementary.pdf]

Table S1. Univariate and Multivariate Analyses for risk factors for parasitic infections in the study population.

			Univariate analysis			Multivariate analysis		
	**Tested positive for parasitic infection (n/N)**	**Tested positive for parasitic infection (%)**	**Odds Ratio**	**95% Confidence Interval**	**P-value**	**Odds Ratio**	**95% Confidence Interval**	**P-value**
**Gender**								
Male	392/1420	27.61	1					
Female	248/965	25.70	0.907	0.753-1.092	0.303			
**Age class (years)**								
0–4	220/1006	21.87	1					
5–9	349/1123	31.08	1.618	1.330-1.967	<0.0001	1.540	1.247-1.878	<0.0001
10–14	67/227	29.52	1.496	1.084-2.064	0.014	1.456	1.042-2.034	0.028
15–18	4/29	13.79	0.572	0.197-1.660	0.304	0.555	0.189-1.629	0.284
**Region of Origin**								
Eastern Europe	204/955	21.36	1					
Africa	126/409	30.81	1.639	1.262 2.127	<0.0001	1.727	1.323-2.254	<0.0001
Latin America	185/529	34.97	1.980	1.563 2.508	<0.0001	1.787	1.402 2.275	<0.0001
Asia	125/492	25.41	1.254	0.971-1.618	0.082	1.238	0.952 1.610	0.112
**Eosinophil count (cell/mcL)**								
≤500	464/1915	24.23	1					
501–999	127/339	37.46	1.873	1.468-2.389	<0.0001	1.823	1.423-2.334	<0.0001
≥1000	41/87	47.13	2.787	1.806-4.301	<0.0001	2.628	1.689-4.088	<0.0001
Missing	8/44	18.18	0.695	0.321-1.506	0.356	0.670	0.306 1.465	0.315
**Anemia**								
No	444/1577	28.15	1					
Yes	54/185	29.19	1.052	0.752-1.471	0.768			
Missing	142/623	22.79	0.753	0.606–0.936	0.011			
**Vitamin D status**								
Deficiency	165/597	27.64	1					
Severe Deficiency	54/187	28.88	1.063	0.740-1.529	0.742			
Insufficient	206/670	30.75	1.162	0.911-1.482	0.225			

Normal	129/539	23.93	0.824	0.637-1.076	0.155
Missing	86/392	21.94	0.736	0.546–0.992	0.044

Table S2. Univariate and Multivariate analyses for risk factors for a positive test result for at least one pathogen parasite

	Eosinophilia (n/N)	Eosinophilia (%)	Univariate analysis			Multivariate analysis		
			Odds Ratio	95% Confidence Interval	P-value	Odds Ratio	95% Confidence Interva	P-value
**Gender**								
Male	267/1396	19.13						
Female	159/945	16.83	0.855	0.689-1.062	0.157			
**Age Class (Years)**								
0–4	163/989	16.48	1					
5–9	220/1099	20.02	1.268	1.014-1.586	0.037	1.010	0.677-1.509	0.958
10–14	39/225	17.33	1.062	0.724-1.560	0.757	0.923	0.312-2.729	0.885
15–18	4/28	14.29	0.845	0.289-2.467	0.757	1.147	0.906-1.453	0.255
**Region of Origin**								
Eastern-Europe	140/942	14.86	1					
Africa	66/406	16.26	1.112	0.808-1.530	0.514	1.175	0.844-1.637	0.33
Latin-America	108/517	20.89	1.513	1.146-1.997	0.004	1.356	1.017-1.807	0.038
Asia	112/476	23.53	1.762	1.335-2.327	<0.0001	1.872	1.406-2.494	<0.0001
**Anemia**								
No	275/1576	17.45	1					
Yes	43/183	23.50	1.453	1.008–2.094	0.045			
Missing	108/582	18.56	1.078	0.843-1.379	0.550			
**Vitamin D Status**								
Deficiency	107/597	17.92	1					
Severe Deficiency	38/184	20.65	1.191	0.788-1.802	0.406			
Insufficient	115/667	17.24	0.954	0.714-1.275	0.751			
Normal	100/531	18.83	1.062	0.785-1.437	0.694			
Missing	66/362	18.23	1.021	0.727-1.433	0.904			
**Parasitic Infection**								
None	258/1709	15.10	1					

Protozoon	62/315	19.68	1.378	1.013-1.874	0.941	0.771	0.564-1.056	0.105
Helminth	83/260	31.92	2.637	1.968-3.533	<0.0001	2.061	1.391-3.056	<0.0001
Protozoon plus Helminth	23/57	40.35	3.804	2.205-6.564	<0.0001	2.742	1.498-5.017	0.001
